# Immunization against *Clostridium perfringens *cells elicits protection against *Clostridium tetani *in mouse model: identification of cross-reactive proteins using proteomic methodologies

**DOI:** 10.1186/1471-2180-8-194

**Published:** 2008-11-11

**Authors:** Syed Imteyaz Alam, Sunita Bansod, Lokendra Singh

**Affiliations:** 1 Biotechnology Division, Defence Research & Development Establishment, Gwalior 474002, India

## Abstract

**Background:**

*Clostridium tetani *and *Clostridium perfringens *are among the medically important clostridial pathogens causing diseases in man and animals. Several homologous open reading frames (ORFs) have been identified in the genomes of the two pathogens by comparative genomic analysis. We tested a likelihood of extensive sharing of common epitopes between homologous proteins of these two medically important pathogens and the possibility of cross-protection using active immunization.

**Results:**

Eight predominant cross-reactive spots were identified by mass spectrometry and had hits in the *C. tetani *E88 proteome with significant MOWSE scores. Most of the cross-reactive proteins of *C. tetani *shared 65–78% sequence similarity with their closest homologues in *C. perfringens *ATCC13124. Electron transfer flavoprotein beta-subunit (CT3) was the most abundant protein (43.3%), followed by methylaspartate ammonia-lyase (36.8%) and 2-phosphoglycerate dehydratase (35.6%). All the proteins were predicted to be cytoplasmic by PSORT protein localization algorithm. Active immunization with *C. perfringens *whole cells elicited cross-protective immunity against *C. tetani *infection in a mouse model.

**Conclusion:**

Most of the dominant cross-reactive proteins of *C. tetani *belonged to the cluster of orthologous group (COG) functional category, either of posttranslational modification, protein turnover, and chaperones (O) or energy production and conversion (C). The homologs of the identified proteins have been shown to play role in pathogenesis in other Gram-positive pathogenic bacteria. Our findings provide basis for the search of potential vaccine candidates with broader coverage, encompassing more than one pathogenic clostridial species.

## Background

*Clostridium tetani *and *Clostridium perfringens *are among the medically important clostridial pathogens causing diseases in man and animals. *Clostridium tetani *is an anaerobic pathogen possessing a wide arsenal of virulence factors and is the causative agent for tetanus disease. Tetanus disease, and in particular maternal and neonatal tetanus, is still an important cause of death due to insufficient immunization [1,2]. Neonatal tetanus is considered endemic to 90 developing countries and resulted in 248000 deaths in 1997 (World Health Organization; ). Tetanus continues to cause ~250,000 deaths worldwide each year, predominantly in low- and middle-income countries. Tetanus is characterized by muscle rigidity and painful muscle spasms caused by tetanus toxin's blockade of inhibitory neurons that normally oppose and modulate the action of excitatory motor neurons.

On the other hand, *C. perfringens *is an obligate anaerobic rod shaped bacterium commonly found in the gastrointestinal tracts of both animals and humans and widely distributed in soil and sewage. It is an etiological agent, causing several diseases in humans and animals; the former includes gas gangrene, food poisoning, necrotizing enterocolitis of infants and enteritis necroticans [3-5]. The incidence of disease ranged from 1% or less of wounded personnel during World War II to 10% of wounded personnel during World War I. Hundreds of thousands of soldiers died of gas gangrene as a result of battlefield injuries, and *C. perfringens *was widely recognised as being the most important causal organism of the disease.

Many vaccines have been developed from live attenuated forms of bacterial pathogens or from killed bacterial cells [6]. However, an increased awareness of the potential for transient side effects following vaccination has prompted an increased emphasis on the use of subunit vaccines. Despite the fact that a high-level antibody response does not always correlate with protection, presence of antibodies in a host surviving infection can offer clues towards identification of protective antigens of a pathogen.

Several striking findings have emerged from the complete genome sequencing data of these clostridial pathogens [7,8]. Many homologous ORFs have been identified in the genomes of *C. tetani *and *C. perfringens *by comparative genomic analysis of the two genomes. Of the total 2372 ORFs observed in *C. tetani *E88, 1705 ORFs had a close homologue in *C. perfringens *genome showing significant sequence similarity [8]. This suggested a likelihood of extensive sharing of common epitopes between homologous proteins of these two medically important pathogens. To examine this hypothesis, we probed the total cellular proteins of *C. tetani *with antisera raised against whole cells of *C. perfringens *ATCC13124. Cross-reactive proteins have been identified and protection against challenge with *C. tetani *to animals actively immunized with *C. perfringens *whole cell has been reported.

## Results and discussion

Immunization against heat killed *C. perfringens *organisms produced a high titer of antibodies (1:10000) recognizing several proteins as revealed by Western blot analysis of one dimensional SDS-PAGE separated proteins from *C. perfringens *whole cell lysate (data not shown). In contrast serum obtained from sham immunized animals was devoid of such antibody.

**Figure 1:**
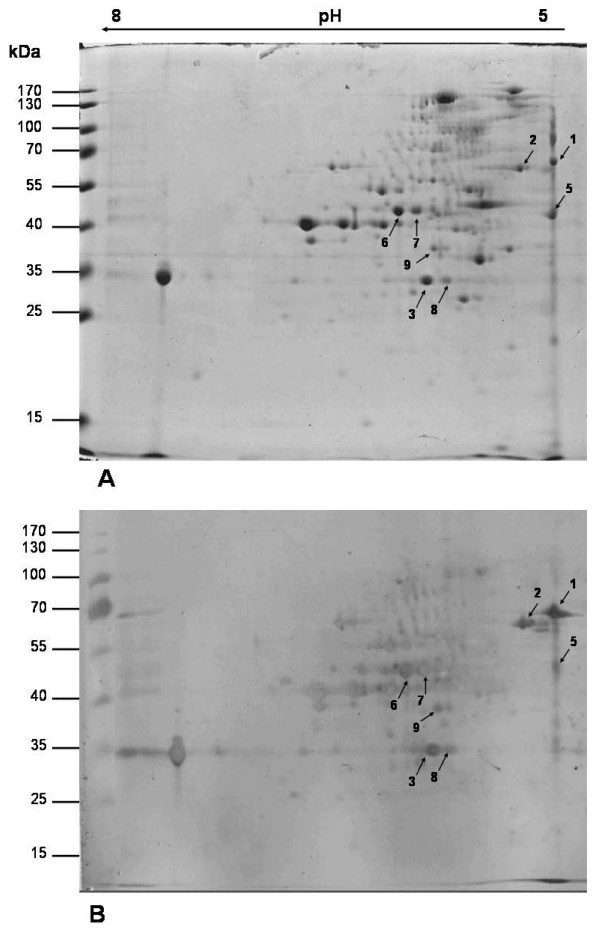
**Two dimensional gel electrophoresis (2-DE) profile of *Clostridium tetani *whole cell lysate **(A) **and immunoblot developed against mice anti-*C. perfringens *(whole cell) serum **(B)**. **Spots identified are indicated by arrows.

Mouse *C. perfringens *whole cell (CPWC) polyclonal antibody reacted with several proteins of *C. tetani *as revealed by 2-DE blot (Fig.1 in additional file 3). *C. tetani *whole cell lysate (CTWC) was also probed with serum from sham-immunized animals; no spots were detected (data not shown). Eight predominant cross-reactive spots were identified in the parallel-run Coomassie-stained gels by use of the coordinates of molecular weight markers and p*I *values on linear IPG strips on the 2DE gel. The spots were excised and identified by mass spectrometry (Table 1 in additional file 1). All the spots had a match in *C. tetani *E88 proteome with significant MOWSE score using the Protein Prospector search tool and the same hits were observed using Mascot search engine with significant protein score. Eight to nineteen peptides were identified in the peptide mass fingerprint and the % coverage for the identified proteins ranged between 33 – 55% except for spot CT7 which remained a little low (27%). On most occasions there was a good correlation between the observed and theoretical molecular mass and p*I *values. The results suggest unambiguous identification of cross-reactive proteins of *C. tetani *by peptide mass fingerprinting. Extensive epitope mapping would be required to identify precise antibody recognition regions of the cross-reactive proteins.

The cross-reactive proteins of *C. tetani *shared 65 – 78% sequence similarity with their closest homologues in *C. perfringens *ATCC13124, except for methylaspartate ammonialyase (spot CT7) which showed 30% sequence identity (Table 1 in additional file1). Most of the dominant cross-reactive proteins belonged to the COG functional category, either of posttranslational modification, protein turnover, and chaperones (O) or energy production and conversion (C) (Table 1 in additional file 1). Electron transfer flavoprotein beta-subunit (CT3) was the most abundant protein (43.3%) followed by methylaspartate ammonia-lyase (36.8%) and 2-phosphoglycerate dehydratase (35.6%). All the proteins were predicted to be cytoplasmic by PSORT protein localization algorithm.

Spot CT1 belonged to the family of Hsp70 chaperones that are known to assist folding of many proteins. It is interesting to note that the hsp70 has been shown to play an important role in imparting adaptive features which allow *Helicobacter pylori *to survive and establish chronic gastroduodenal infections [11-13]. The two cytoplasmic heat shock proteins (HSPs), DnaK and GroEL, have been shown to be among the major targets of the humoral immune response to a *Chlamydia trachomatis *infection [14]. Antibodies to *C. tetani *DnaK and groEL (Table 1 in additional file 1) may have an impact on other infections because HSPs are highly conserved in evolution. Curiously, GroEL has also been implicated in the adherence of *C. difficile *and found to be predominantly cytoplasmic and membrane bound, consistent with surface localization of this protein in clostridia [15]. The 60 kDa form of chaperonin (spot CT2) is also the immunodominant antigen of patients with Legionnaire's disease, and is thought to play a role in the protection of the *Legionella *bacteria from oxygen radicals within macrophages [16]. This hypothesis is based on the finding that the *cpn*60 gene is up-regulated in response to hydrogen peroxide, a source of oxygen radicals. A similar role for protection against oxygen radicals for these clostridial species needs to be explored as generation of anaerobic environment for proliferation of anaerobic clostridial pathogens like *C. perfringens *and *C. tetani *is the first step in colonization of the bacteria.

Two cross-reactive *C. tetani *proteins (spot CT6 and CT7) were identified as methylaspartate ammonialyase having ClpB proteins as a homolog in the *C. perfringens *ATCC13124 proteome. Clp amino terminal domain is found in one or two copies at the amino terminus of ClpA and ClpB proteins from bacteria and eukaryotes. The function of these domains is uncertain but they have been postulated to form a protein binding site. The proteins are thought to be subunits of ATP-dependent proteases which act as chaperones to target the proteases to substrates [17,18]. These proteins are part of the complex program that bacteria undergo in order to survive adverse environmental conditions. Interestingly, the proteins under the control of the regulatory two-component system VirR/VirS in *C. perfringens *have been analyzed by using two-dimensional gel electrophoresis of the culture supernatant from the wild type and the *virR *mutant. Based on MALDI-TOF mass spectrometry, seven positively regulated proteins and eight negatively regulated proteins were identified that included ClpB protein [19].

Enolase (spot CT5) and butyrate kinase (spot CT9) have been observed as cross-reactive protein of *C. tetani *(Table 1 in additional file 1, Fig. 1). Streptococcal enolase has been implicated in GAS adherence to and invasion of human pharyngeal cells and is a highly immunogenic autoantigen with a possible role in the initiation of post-streptococcal sequelae [20]. Curiously, butyrate kinase pathway among butyrate-producing bacteria from the human colon has been shown to have restricted distribution. Butyryl-CoA:acetate CoA-transferase activity was detected in all 38 strains examined, suggesting that it, rather than butyrate kinase, provides the dominant route for butyrate formation in the human colonic ecosystem that contains a constantly high concentration of acetate [21].

Survival of animals that were immunized against heat killed *C. perfringens *organism and were challenged with *C. tetani *viable cells has been shown in table 2 in additional file 2. Except for the lowest dose of challenge (10^2 ^cfu), most of the deaths were recorded within 30 h of challenge and survival did not decrease further during the remaining 5 days of study. In most of the cases, paralysis of limbs preceded death. Immunization with *C. perfringens *did not result in protection against a direct tetanus toxin challenge when tested with 1, 3 and 5 MLD of crude tetanus toxin preparations, indicating a non-tetanus-toxin based process of protection, inhibiting colonization of the bacterium. We did not observe any binding of antibodies from polyclonal serum with tetanus toxin when tested on a western blot using 10 μg of recombinant TeNT. The complete ORF of TeNT was previously cloned and expressed in pQE30 vector and the recombinant protein purified using Ni-NTA column (unpublished results).

Ability of the mice *C. perfringens *whole cell (CPWC) polyclonal antibody to bind on the surface of the *C. perfringens *and *C. tetani *cells was tested using anti-IgG FITC conjugate as described in the methods. A bright yellow green fluorescence was observed, with whole cells of both *C. perfringens *and *C. tetani *(Additional file 4) indicating a surface binding of the mouse polyclonal antibody, raised against heat-killed *C. perfringens *cells. The corresponding slide where un-immunised serum was used, did not exhibit a detectable fluorescence.

Despite a predicted cytoplasmic localization of the identified proteins, their exact location needs to be experimentally verified. As immuno-fluorescence microscopy indicated binding of the polyclonal CPWC antibody to the surface of both *C. perfringens *and *C. tetani *(Additional file 3), we do not rule out the possibility of at least some of the cross-reactive proteins identified in the present investigation, to be actually surface localized. This could be a possible explanation for the cross-protection observed against *C. tetani *cells. However, other low abundance cross-reactive proteins not identified here, remains another possibility. A more detailed study, using monoclonal antibodies and identification of additional cross-reactive proteins is required to ascertain the exact basis of the cross-protection. It is also possible that some antibodies, presumably protective in nature and recognize other proteins, has not been detected in our study. The proteomic approach used in this study identified only cross-reactive proteins shared between *C. perfringens *and *C. tetani*. It remains possible that other (non protein) antigens are also shared between these two bacteria and that those shared antigens contribute to the protection observed when *C. perfringens*-immunized mice are challenged with *C. tetani*.

Only little is known about protein secretory systems in clostridia. In a previous study, the culture supernatant of *C. perfringens *at the late exponential growth phase was shown to contain intracellular proteins that had no putative signal sequences, such as ribokinase, β-hydroxybutyryl-coenzyme A dehydrogenase, fructosebisphosphate aldolase, and elongation factor G [19]. In other studies also, a significant number of cytoplasmic proteins have been identified as cell-wall associated proteins/immunogens [22,23]. The normally cytoplasmic chaperonins DnaK and GroEL have been identified as immunoreactive surface antigens of *S. pyogenes *[22]. In another report, 21 predicted cytoplasmic proteins were detected on the surface of *S. pyogenes *[23]. Though authors did not address the mechanism by which proteins localize to the cell surface, several lines of evidence suggested that no measurable lysis occurred during the treatment of the cells with trypsin. Since a signal peptide and LPXTG motif were absent, it was suggested that these proteins are either passively released during autolysis or that an alternative secretory pathway may exist for many secreted GAS proteins.

## Conclusion

Most of the dominant cross-reactive proteins of *C. tetani *belonged to the COG functional category, either of posttranslational modification, protein turnover, and chaperones (O) or energy production and conversion (C). The homologs of the identified proteins have been shown to play a role in pathogenesis in other Gram-positive pathogenic bacteria. Our findings provide basis for the search of potential vaccine candidates with broader coverage, encompassing more than one pathogenic clostridial species. They also generate curiosity to explore any correlation of these results with the general immunity and success of immunization programmes against *C. tetani*; in developed countries, tetanus is little more than a medical curiosity. It has to be borne in mind that the primary habitat of *C. tetani *is soil and unlike *C. perfringens*, is not commonly found in the gastrointestinal tracts of animals and humans. The cross-protective nature of *C. perfringens *could be a possible reason as to why neonatal tetanus is more prevalent than tetanus disease in adults having extensive colonization of and exposure to *C. perfringens*.

## Methods

### Bacterial strains and growth conditions

The clinical strain of *Clostridium tetani *was obtained from Christian Medical College, Vellore, India. The strain exhibited 100% sequence identity with *C. tetani *strain E88 at 16S rDNA level. *Clostridium perfringens *ATCC13124 was obtained from Becton Dickinson India Pvt. Ltd., India. The clostridial isolates were cultivated anaerobically at 37°C in trypticase peptone yeast-extract glucose (TPYG) broth containing pancreatic digest of casein, 50 g; peptone, 5 g; yeast extract, 20 g; glucose, 4 g; sodium thioglycollate, 1 g; cycloserine, 250 mg; sulphamethoxazole, 76 mg and trimethoprim, 4 mg per litre.

### Immunization and protection studies

Animal experiments were approved by the institutional Animal Ethical Committee at DRDE, Gwalior. Four-week-old female BALB/c mice were actively immunized against heat-killed vegetative cells of *C. perfringens *in a four week immunization schedule. Cells were grown in TPYG broth at 37°C, harvested in the exponential phase (OD_600 nm _0.8–1.0) and washed with phosphate buffer saline (PBS). The number of bacteria in the final suspension was determined by plating 10-fold serial dilutions onto SPS agar (Difco, USA) plates containing tryptone, 15 g; yeast extract, 10 g; ferric citrate, 0.5 g; sodium sulfite, 0.5 g; sodium thioglycollate, 0.1 g; polysorbate 80, 0.05 g; sulfadiazine, 0.12 g; polymyxin B sulfate, 0.01 g; agar, 15 g per litre. Heat inactivation was accomplished in a water bath at 60°C for 30 min. No live bacteria were detected after this suspension was plated onto agar plates. Cells were injected intraperitoneally using Freund's complete adjuvant (Sigma Aldrich, India) for the first immunization and Freund's incomplete adjuvant for booster immunizations. On day 1 and 7, 10^2 ^cfu (100 μl cell suspension in PBS and 100 μl adjuvant) was injected in each mouse while on day 14 and 27 the dose was increased to 10^4 ^CFU. One week after administration of the last booster, 10 animals were anesthetized by halothane inhalation, and blood specimen (500 μl) was obtained from each by means of retroorbital puncture. Serum from these specimens was pooled and was used to verify specific antibody production by Western blot analysis. Sham-immunized animals received an equal volume of adjuvant alone in a parallel, same immunization schedule and serum was collected after 5 weeks.

For protection studies, four-week-old female BALB/c mice were similarly immunized with heat killed vegetative cells of *C. perfringens *in a four week immunization schedule as described above. On day 37, groups of immunized (with CPWC) and sham-immunized animals (n = 12) were challenged intra-peritonealy with different inocula of freshly grown *C. tetani *cells (1.2 × 10^2^, 1.2 × 10^4^, 1.2 × 10^6^, and 1.2 × 10^8 ^cfu). Death, as well as paralytic symptoms were recorded every 6 hr up to day 2 and every 12 hours for seven days. Animals that survived for the entire 7-days were considered survivors.

The culture supernatant from *C. tetani *was titrated for estimation of minimum lethal dose (MLD) by serial two-fold dilutions made in gelatin diluent. One half milliliter of each dilution was inoculated i.p. into each of six 20 gram mice. The mice were observed for four days and the flaccid paralysis and deaths were recorded. Mice immunized with heat killed *C. perfringens*, were challenged with 1, 3, and 5 MLD (n = 6) of crude tetanus toxin and observed for death and paralysis. Sham-immunised mice (n = 6) were also given equivalent doses of tetanus toxin i.p.

### Two dimensional gel electrophoresis (2-DE)

Twenty milliliter of culture was harvested in the exponential growth phase (OD_600 nm_~0.8) and washed in 50 mM Tris/HCl, pH 7.5. The cells were resuspended in the same buffer supplemented with protease inhibitor (Protease inhibitor cocktail, Sigma). Cell lysis was performed by sonication and the un-disrupted cells were removed by centrifugation (6000 × g; 15 min; 4°C). In order to improve focusing of proteins the lysates (500 microgram) were purified using 2D-cleanup kit (Bio-Rad) and the protein pellet was finally resuspended in 250 μl of sample rehydration buffer (8 M urea, 2% w/v CHAPS, 15 mM DTT and 0.5% v/v IPG buffer pH 3–10).

Total protein concentration was determined according to the method of Bradford [9] using Quick Start Bradford Protein Assay kit (Bio-Rad, USA) as per manufacturer's instructions. The protein concentration was calculated using bovine serum albumin (BSA) as standard.

The isoelectric focusing was performed using immobilized pH gradient (IPG) strips (Bio-Rad, USA). IPG strips with a pH range from 5–8 were used for all the experiments. For the first dimension 250 μg of protein samples in 125 μl of rehydration solution was used to rehydrate IPG strip (7 cm, pH 5–8). The IPG strips were rehydrated overnight and then the proteins were focused for 8000 VHr at 20°C under mineral oil. After focusing, the strips were incubated for 10 min, in 2 ml of equilibrium buffer I (6 M urea, 30% w/v glycerol, 2% w/v SDS and 1% w/v DTT in 50 mM Tris/HCl buffer, pH 8.8) followed by equilibrium buffer II (6 M urea, 30% w/v glycerol, 2% w/v SDS and 4% w/v iodoacetamide in 50 mM Tris/HCl buffer, pH 8.8). After the equilibration steps the strips were transferred to 12% SDS-PAGE for the second dimension by the method of Blackshear [10]. Protein spots were visualized by staining with Commassie Brilliant Blue G-250. Gel images were capture by GS800 densitometer (Bio-Rad, USA). Relative abundance of spots was determined by PD Quest software (Bio-Rad, USA).

Two independent cell preparations were pooled together for running the 2DE gel and obtaining the corresponding immunoblot. Replicate gels were generated from two such independent experiments, of which one representative gel has been shown.

### Immunoblotting

For immunoblotting, the one dimensional or 2-DE separated proteins were transferred electrophoretically to nitrocellulose membrane (Bio-Rad, Hercules, CA) and then blocked with 5% skim milk in PBS (pH 7.2). Hyper-immune serum from mice raised against *C. perfringens *whole cell (CPWC) was used at 1:4000 dilutions in blocking buffer. Goat anti-mouse HRP conjugate was used as secondary antibody and blots were developed using the Immuno-Blot HRP assay kit (Bio-Rad, USA) as per manufacturer's instructions.

### Immunofluorescence Microscopy

One milliliter of culture was harvested in the exponential growth phase (OD_600 nm_~0.8), washed in phosphate buffer saline (PBS), pH 7.2, and resuspended in 1 ml of same buffer. Twenty microlitre of cells were air dried on 4 mm wells of printed slides (Flow Lab, USA) and fixed with methanol for 5 min. *C. perfringens *ATCC13124 and *C. tetani *cells were added to duplicate wells on two independent slides. Each spot was washed twice with PBS and blocked with 0.5% gelatin in PBS (PBSG) for 30 min at 37°C. To one slide, serum from sham-immunized mice (40 μl) was added at 1:40 dilution in PBSG and to the other slide mouse anti-*C. perfringens *serum was similarly added. Slides were incubated in humid chamber at 37°C for 1 hr and then washed 4 times with PBS. Forty microlitre of goat anti-mouse IgG fluorescein isothiocyanate (FITC) conjugate (Dako, Denmark) was added (1:20 diluted in PBSG) to each well and incubated in dark, moist chamber for 30 min at 37°C. Slides were washed with 4 changes of PBS and observed under a fluorescence microscope (Leica, Germany).

### Identification of protein spots by mass spectrometry

Protein spots were excised with the help of thin-walled PCR tubes (200 μl) appropriately cut at the bottom with the help of fresh surgical scalpel blade. Care was taken not to contaminate the spots from adjoining proteins or with skin keratin. The gel spots were washed with proteomic grade de-ionized water and identified by mass spectrometry by the commercial services provided by Dominion Pharmakine, Spain. The gel piece containing the protein was destained, reduced/alkylated and digested using the Montage In-Gel Digest Kit (Millipore) following the kit's instructions.

Analysis was performed using the Voyayer-DE PRO BioSpectrometry workstation from Applied Biosystems. Spectrum was obtained in the mass range of 500–4000 Da and calibrated using a calibration mixture containing angiotensin I, Substance P, ACTH (1–17), ACTH (18–39) and somatostain (28). Peptides masses of the unknown proteins were sent to two different peptide mass fingerprinting databases, Mascot from Matrix Science  and MS-Fit from Protein Prospector  and/or Aldente . There were 3292813 entries in the Mascot database at the time of search. Search parameters were as follow: maximum allowed peptide mass error of 100 ppm, consideration of one incomplete cleavage per peptide and at least 4 peptides identified. Protein localization prediction was performed by the online tool PSORT at .

## Authors' contributions

SIA designed and executed most part of the experiments including proteomic studies and challenge experiments. SB participated in running 2DE gels and immunisation of animals. LS provided supervision of the research group and critically revised the manuscript for its important intellectual content.

## Supplementary Material

Additional file 1 Click here for file

Additional file 2 **Table 2** Click here for file

Additional file 3 **Replicate 2DE gels stained with coomassie brilliant blue [A (i) and (ii)] and corresponding immunoblots [B (i) and (ii)] developed against mice anti-*C. perfringens *(whole cell) serum.** Click here for file

Additional file 4 ***C. perfringens*****(A) *****and *C. *tetani*****(B) cells showing immuno-fluorescence under fluorescent microscope(magnification-100 ×).** Cells were allowed to bind with mouse polyclonal anti-*C. perfringens *(whole cell) serum and binding was revealed by goat anti-mouse IgG-FITC conjugate. **C**, Control image of *C. perfringens *cells which were allowed to bind with sham-immunized serum followed by addition of revealing anti-mouse IgG-FITC conjugate. Click here for file
