# Supplementary material for: Immunization against Clostridium perfringens cells elicits protection against Clostridium tetani in mouse model: identification of cross-reactive proteins using proteomic methodologies
Source: BMC Microbiol. 2008 Nov 11;8:194. doi: 10.1186/1471-2180-8-194 (PMC2621373; doi:10.1186/1471-2180-8-194)
Supplement: Additional file 1 [file 1471-2180-8-194-S1.doc]

**Table 1:** Cross-reactive proteins of *C. tetani* developed against mice anti *C. perfringens* whole cell serum.. The table reports: 1) the gene locus and protein function of the MS-Fit top hit with MOWSE score and percent coverage, 2) observed and experimental M_r_ / pI values 3) homologous protein in *C. perfringens* ATCC13124 proteome with percent identity, 4) predicted cellular localization of proteins and relative abundance.

| **Spot #** | **MS-Fit (Protein Prospector) top hit^a^** | **MOWSE**  **Score** | **% coverage** | **M_r_ / p*I*_theoretical_** | **M_r_ / p*I*_observed_** | **Homolog in *C. perfringens* ATCC13124^b^** | **% identity** | **Protein localization** | **Relative abundance^c^ (%)** |
| --- | --- | --- | --- | --- | --- | --- | --- | --- | --- |
| CT1 | Chaperone protein dnaK, *C. tetani* E88 (gi|28211653) | 3.41e+13 | 47.9 | 66394 / 4.7 | 66500 / 5.0 | Chaperone protein DnaK ([Q0TNS7](http://www.expasy.org/uniprot/Q0TNS7)), **O** [Name=dnaK;] | 78 | Cyt (9.65) | 25.9 |
| CT2 | 60 kDa chaperonin groEL, *C. tetani* E88 (gi|28212000) | 7.85e+10 | 36.1 | 58468/4.8 | 61000 / 5.2 | 60 kDa chaperonin (groEL protein, [CH60](http://www.expasy.org/uniprot/CH60)), **O** [Name=groL] | 77 | Cyt (9.98) | 17.8 |
| CT3 | Electron transfer flavoprotein beta-subunit, *C. tetani* E88 (gi|28212010) | 2.06e+7 | 44.4 | 28261/5.2 | 32000 / 5.8 | Electron transfer flavoprotein, beta subunit / FixA family protein, **C** ([Q0TN17](http://www.expasy.org/uniprot/Q0TN17)) | 65 | Cyt (8.87) | 43.3 |
| CT5 | Enolase (EC 4.2.1.11) (2-phosphoglycerate dehydratase) (2-phospho-Dglycerate  hydro-lyase), *C. tetani* E88 (gi|59797631, ENO_CLOTE) | 7.53e+9 | 36.2 | 46577/4.6 | 41000 / 5.0 | Enolase (2-phosphoglycerate dehydratase, [ENO](http://www.expasy.org/uniprot/ENO)), **G**  [Name=eno;] | 76 | Cyt (9.65) | 35.6 |
| CT6 | Methylaspartate ammonia-lyase, *C. tetani* E88 (gi|28212141) | 9.64e+6 | 33.5 | 45618/5.3 | 44000 / 6.0 | ClpB protein,  [Q0TQH1](http://www.expasy.org/uniprot/Q0TQH1), **O**  [Name=clpB;] | 30 | Cyt (8.87) | 36.8 |

| **Spot #** | **MS-Fit (Protein Prospector) top hit^a^** | **MOWSE**  **Score** | **% coverage** | **M_r_ / p*I*_thepretical_** | **M_r_ /p*I*_observed_** | **Homolog in *C. perfringens* ATCC13124^b^** | **% identity** | **Protein localization** | **Relative abundance^c^** |
| --- | --- | --- | --- | --- | --- | --- | --- | --- | --- |
| CT7 | Methylaspartate ammonialyase, *C. tetani* E88 (gi|28212141) | 570106 | 27.2 | 45618/5.3 | 44000 / 6.0 | ClpB protein,  [Q0TRU7](http://www.expasy.org/uniprot/Q0TRU7), **O** [Name=clpB;] | 30 | Cyt (8.87) | 27.9 |
| CT8 | Electron transfer flavoprotein beta-subunit, *C. tetani* E88 (gi|28212010) | 3.60e+6 | 33.2 | 28261/5.2 | 32000 / 5.7 | Electron transfer flavoprotein, beta subunit/FixA family protein, [Q0TN17](http://www.expasy.org/uniprot/Q0TN17), **C** | 65 | Cyt (8.87) | 13.7 |
| CT9 | Butyrate kinase, *C. tetani* E88 (gi|28212124) | 2.23e+9 | 55.1 | 38902/5.3 | 40000 / 5.8 | Butyrate kinase, [BUK](http://www.expasy.org/uniprot/BUK) (BK), **C**  [Name=buk] | 67 | Cyt (9.98) | 6.0 |

**^a^** Protein accession number and name are shown. MALDI-TOF analysis was performed using the Voyayer-DE PRO BioSpectrometry workstation from Applied Biosystems. Peptides masses of the unknown proteins were sent to two different peptide mass fingerprinting databases, Mascot from Matrix Science (http://www.matrixscience.com) and MS-Fit from Protein Prospector (http://prospector.ucsf.edu) and /or Aldente (http://www.expasy.ch/tools/aldente). Search parameters were as follow: maximum allowed peptide mass error of 100ppm, consideration of one incomplete cleavage per peptide and at least 4 peptides identified. Only, MS-Fit from Protein Prospector results, are shown here.

**^b^** Homologous protein in *C. perfringens* ATCC13124 proteome was searched using SWISS-PROT data base at <http://www.expasy.ch/>. Bold letters indicate functional category at COG data base.

**^c^** Relative to most abundant protein on the gel based on total spot density (OD x mm^2^).
