# Supplementary material for: Immunization against Clostridium perfringens cells elicits protection against Clostridium tetani in mouse model: identification of cross-reactive proteins using proteomic methodologies
Source: BMC Microbiol. 2008 Nov 11;8:194. doi: 10.1186/1471-2180-8-194 (PMC2621373; doi:10.1186/1471-2180-8-194)
Supplement: Additional file 2 — Table 2 [file 1471-2180-8-194-S2.doc]

**Table 2:** Result of *C. tetani* challenge to mice immunized with *C. perfringens* whole cells.

| **Immunization group** | **Challenge dose**  **(*C. tetani* cells i.p.)** | **No. of animals challenged** | **No. of animals that survived^a^ (%)** | **Time to death (hr)** |
| --- | --- | --- | --- | --- |
| *C. perfringens* whole cell | 1.2 x 10^8^ | 10 | 0 (0±0) | 24-30 |
|  | 1.2 x 10^6^ | 12 | 8 (66.6±8.33)* | 160-172 |
|  | 1.2 x 10^4^ | 10 | 10 (100±0)* | - |
|  | 1.2 x 10^2^ | 12 | 12 (100±0)* | - |
| Adjuvant control | 1.2 x 10^8^ | 12 | 0 (0±0) | 10-12 |
|  | 1.2 x 10^6^ | 12 | 0 (0±0) | 20-24 |
|  | 1.2 x 10^4^ | 10 | 0 (0±0) | 20-24 |
|  | 1.2 x 10^2^ | 12 | 4 (33±8.33) | 150-162 |

**^a^** Each challenge dose was given to 3 groups of 3-4 animals and percent survival values are mean ± standard error.

* Significantly different from control by student’s T-test at P≤0.001.
