# Supplementary figures and images for: Immunization against Clostridium perfringens cells elicits protection against Clostridium tetani in mouse model: identification of cross-reactive proteins using proteomic methodologies
Source: BMC Microbiol. 2008 Nov 11;8:194. doi: 10.1186/1471-2180-8-194 (PMC2621373; doi:10.1186/1471-2180-8-194)

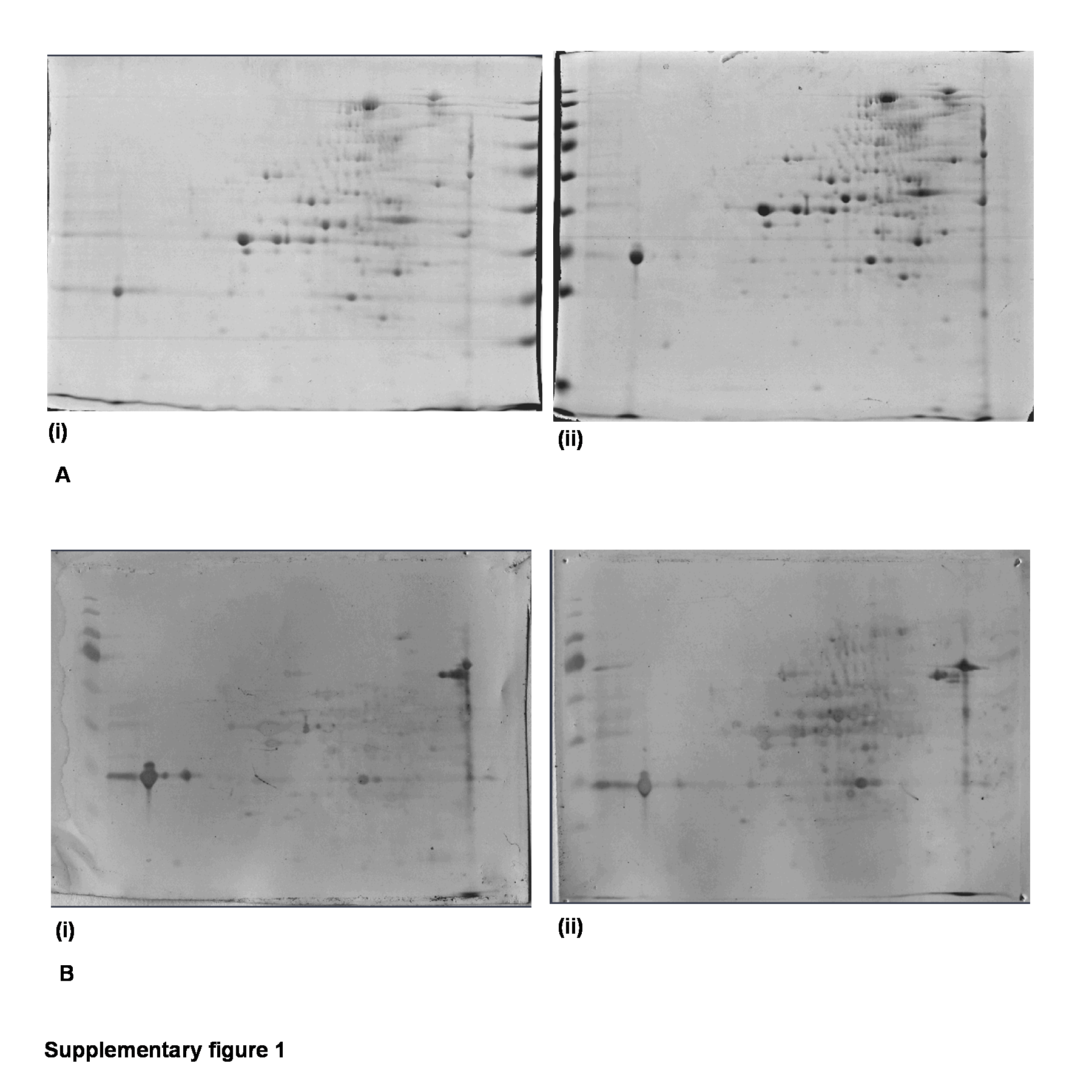

Supplement: Additional file 3 — Replicate 2DE gels stained with coomassie brilliant blue [A (i) and (ii)] and corresponding immunoblots [B (i) and (ii)] developed against mice anti-C. perfringens (whole cell) serum. [file 1471-2180-8-194-S3.tiff]

## Slide 1
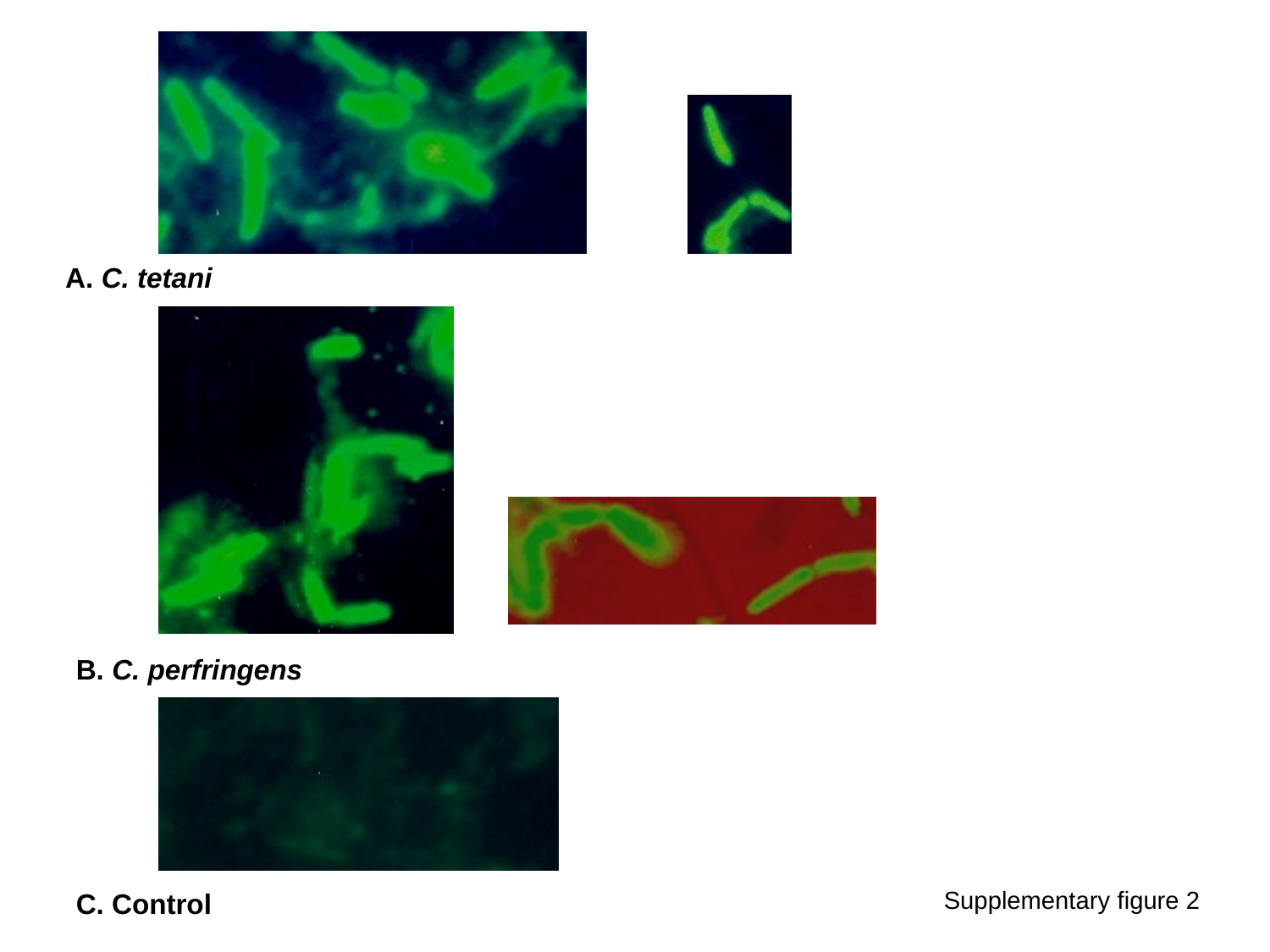

A. C. tetani
B. C. perfringens
Supplementary figure 2
C. Control

Supplement: Additional file 4 — C. perfringens(A) and C. tetani(B) cells showing immuno-fluorescence under fluorescent microscope(magnification-100 ×). Cells were allowed to bind with mouse polyclonal anti-C. perfringens (whole cell) serum and binding was revealed by goat anti-mouse IgG-FITC conjugate. C, Control image of C. perfringens cells which were allowed to bind with sham-immunized serum followed by addition of revealing anti-mouse IgG-FITC conjugate. [file 1471-2180-8-194-S4.ppt]
